# One microRNA has the potential to target whole viral mRNAs in a given human coronavirus

**DOI:** 10.3389/fmicb.2022.1035044

**Published:** 2022-11-10

**Authors:** Tielong Xu, Long-xue Li, Yao Jia, Qingni Wu, Weifeng Zhu, Zhou Xu, Bin Zheng, Xuexin Lu

**Affiliations:** ^1^Evidence-Based Medicine Research Center Department, Jiangxi University of Chinese Medicine, Nanchang, China; ^2^Laboratory Animal Science and Technology Center, Jiangxi University of Chinese Medicine, Nanchang, China; ^3^National Institute of Parasitic Diseases Chinese Center for Disease Control and Prevention, and WHO Collaborating Center for Tropical Diseases, Shanghai, China; ^4^National Institute for Viral Disease Control and Prevention, Chinese Center for Disease Control and Prevention, Beijing, China

**Keywords:** human coronaviruses, microRNA, mRNA, antivirus, SARS-CoV-2

## Abstract

MicroRNAs (miRNAs) can repress viral replication by targeting viral messenger RNA (mRNA), which makes them potential antiviral agents. The antiviral effects of miRNAs on infectious viruses have been explored extensively; however, recent studies mainly considered the action modes of miRNAs, neglecting another key factor, the molecular biology of viruses, which may be particularly important in the study of miRNA actions against a given virus. In this paper, the action modes of miRNAs and the molecular biology of viruses are jointly considered for the first time and based on the reported roles of miRNAs on viruses and human coronaviruses (HCoVs) molecular biology, the general and specific interaction modes of miRNAs-HCoVs are systematically reviewed. It was found that HCoVs transcriptome is a nested set of subgenomic mRNAs, sharing the same 5′ leader, 3′ untranslated region (UTR) and open reading frame (ORF). For a given HCoV, one certain miRNA with a target site in the 5′ leader or 3’ UTR has the potential to target all viral mRNAs, indicating tremendous antiviral effects against HCoVs. However, for the shared ORFs, some parts are untranslatable attributed to the translation pattern of HCoVs mRNA, and it is unknown whether the base pairing between the untranslated ORFs and miRNAs plays a regulatory effect on the local mRNAs where the untranslated ORFs are located; therefore, the regulatory effects of miRNAs with targets within the shared ORFs are complicated and need to be confirmed. Collectively, miRNAs may bepromising antiviral agents against HCoVs due to their intrinsically nested set of mRNAs, and some gaps are waiting to be filled. In this review, insight is provided into the exploration of miRNAs that can interrupt HCoVs infection.

## Introduction

MicroRNAs (miRNAs) are noncoding, 18 ~ 22-nt-long RNAs that function in the posttranscriptional process of gene expression through complementary base pairing with target messenger RNAs (mRNAs; Kim et al., 2009). Many studies based on the action modes of miRNAs have been performed to explore their antiviral effects on various infectious viruses (Scheel et al., 2016; Nieder-Rohrmann et al., 2017; Khan et al., 2020). Now, with a deepened understanding of the biology of viruses, the exploration of miRNAs as antiviral agents should take both factors into consideration (not solely the action modes of miRNAs). In fact, the dual considerations may be essential for a successful exploration to use miRNA as an antiviral agent for a given virus, such as human coronaviruses (HCoVs), as discussed in this review.

CoVs are viruses with a corona-like morphology, with each species assembled similarly by a single-stranded RNA genome, several structural transmembrane glycoproteins, i.e., S, E, M, N, (Supplementary Figure S1) and possibly one or more virus-specific accessory protein (s), e.g., hemagglutinin esterase glycoprotein in HCoV-OC43 (Hartenian et al., 2020). CoVs can be spread through molecules expressed from the respiratory tract or through direct contact. Generally, CoVs are silently carried by wild animals, e.g., bats, camels, and palm civets. Occasionally, due to their ability to quickly evolve, CoVs may spill over into domestic animal and human populations; subsequently, cross-species transmission may occur (de Wit et al., 2016). To date, seven CoVs have crossed species barriers and caused transmission in the human population (Hidalgo et al., 2021; Miranda et al., 2021), termed HCoVs. Four HCoVs, HCoV-229E, NL63, OC43, and HKU1, have evolved to stably circulate in the human population, generally causing the self-limiting common cold (Hamre and Procknow, 1966; Phelan et al., 2020). In the last two decades, three HCoVs resulting in severe respiratory disease have emerged: severe acute respiratory syndrome coronavirus (SARS-CoV) in 2003 (Chan-Yeung and Xu, 2003), Middle East respiratory syndrome coronavirus (MERS-CoV) in 2012 (Chan et al., 2015), and severe acute respiratory syndrome coronavirus 2 (SARS-CoV-2) in 2019 (Xu et al., 2020). Since their emergence, the disease caused by HCoVs is no longer limited to the common cold. The mortality rates of infection can reach approximately 11% for SARS-CoV (Chan-Yeung and Xu, 2003), 35% for MERS-CoV (Chan et al., 2015) and 5% for SARS-CoV-2 (Xu et al., 2020). As of October 12, 2022, SARS-CoV-2 has caused approximately 61.98 million human infections and 6.54 million deaths, and these levels continue to increase (World Health Organization, 2022). On the basis of the three lethal HCoVs documented in the past two decades, HCoVs seem to have periodically emerged and caused outbreaks in the human population (Miranda et al., 2021). Under these conditions, effective tools are urgently needed to interrupt the current and possible future epidemics caused by HCoVs. In this paper, the general interaction modes of miRNAs-viruses and the specific interaction modes of miRNAs-HCoVs corresponding to their molecular biology are jointly reviewed, providing insight into the exploration of miRNAs as antiviral agents against HCoVs.

## General interaction modes of miRNAs-infectious viruses

### Intracellular or extracellular miRNAs can target mRNAs to regulate gene expression

Diverse miRNAs are expressed in organisms and encoded by the genes of plants, animals, parasites, bacteria, and viruses. Animals are hosts of HCoVs, so this paper focuses on the biogenesis of miRNAs in animals. Briefly, the miRNA gene is first transcribed into primary miRNA (pri-miRNA) by RNA polymerase II (Pol II). Pri-miRNA are spliced by Drosha and DGCR8 complex, converting into precursor miRNA (pre-miRNA) in the cellular nucleus. Then, facilitated by exportin 5, pre-miRNA is exported to the cytoplasm, where it is further cleaved by Dicer enzyme into a mature miRNA:miRNA* duplex. The mature miRNA:miRNA* duplex integrates into the miRNA-induced silencing complex (miRISC) combined with AGO, forming an intermediate complex called pre-miRISC. Then, miRNA* is unwound from miRNA, forming functional miRISCs in the cytoplasm (Kim et al., 2009). Apart from intracellular biogenesis, it has been reported that miRNAs can also be imported from extracellular exosomes *via* the circulatory system as free miRNAs or AGO-bound miRNAs (Gebert and MacRae, 2019). Free miRNAs or AGO-bound miRNAs can assemble with the other components of miRISCs to form functional miRISCs and regulate the translation of target mRNAs in receptor cells (Zhang et al., 2012; Zhou et al., 2020), which makes sense for the usage of a miRNA as a tool to interrupt viral infection (Supplementary Figure S2).

The uploaded miRNA guides the miRISC complex to its target (mRNA) *via* complementary base-pairing. Full-length base-pairing between the miRNA and mRNA is not necessary, and only 6 ~ 8 bases at 5′ end of the miRNA at the 2–8 positions are enough for a miRNA to identify its target mRNA (Kim et al., 2009). MiRNAs were initially found to target the 3′ untranslated region (UTR) of mRNA, resulting in inhibition or repression of translation. However, through in-depth ongoing research, targeting sites of miRNAs were also identified in 5’ UTRs (Nieder-Rohrmann et al., 2017) and open reading frames (ORFs; Forman et al., 2008) of mRNA. Additionally, the regulatory effects of miRNAs are multidirectional, including complete degradation of mRNA (Zhang et al., 2018), translation attenuation by suspension of translation (Liu et al., 2005), and conversely upregulated expression of mRNA (Machlin et al., 2011; Scheel et al., 2016; Nieder-Rohrmann et al., 2017; Supplementary Figure S2).

### miRNAs can inhibit viral replication by targeting host or viral mRNAs

MiRNAs can play an important role in viral life *via* posttranscriptional regulation (Sato et al., 2021). The information about the regulatory effects of miRNAs on various viruses has been collected from 176 reported articles and is summarized in Table 1 and Supplementary Table S1, which include the miRNA name, target type, location of the target site in a mRNA, and effect on the target mRNA and ultimate effect on viruses. It is obvious that viral mRNAs retain target sites for miRNAs (Skalsky and Cullen, 2010), and that miRNAs can achieve the regulatory roles by targeting host mRNAs and/or viral mRNAs. The regulatory roles played by miRNAs include inhibiting or promoting viral replication and certain other pathobiology related effects (Xu et al., 2021).

**Table 1:** The reported effects of miRNAs on viruses at different target locations.

Type of target	Effect on virus infection			Total (*n*)	Antiviral ratio (%)
	Inhibiting (*n*)	Promoting (*n*)	Others (*n*)		
Host mRNA	63	62	19	144	43.75
3’ UTR	58	58	17	133	43.61
5’ UTR	0	0	1	1	–
ORF	1	2	0	3	–
Unknown	4	2	1	7	–
Virus mRNA	47	8	1	56	83.93
3’ UTR	17	3	1	21	80.95
5’ UTR	3	4	0	7	42.86
ORF	27	1	0	28	96.43

### miRNAs targeting viral mRNAs exhibit a higher antiviral ratio than miRNAs targeting host mRNAs

The data presented in Table 1 and Supplementary Table S1 shows that miRNAs can interrupt viral replication and that miRNAs targeting viral mRNAs are ideal candidates for antiviral agents. Compared with the miRNAs targeting host mRNAs, the miRNAs targeting viral mRNAs always hold the following advantage. The interaction modes of miRNAs with viral mRNAs seem to be direct and predictable; in contrast, the interaction modes of miRNAs with host mRNAs appear to be indirect and uncertain. As noted in Table 1, most (forty-seven of fifty-six) miRNAs targeting viral mRNAs have been reported to inhibit viral replication, with a few (eight of fifty-six) promoting viral replication. In other words, the antiviral effect of miRNAs targeting viral mRNAs is predictable, with an estimated probability as high as 83.93% (47/56). However, the antiviral effects of miRNAs targeting host mRNAs are less predictable, with only a 43.75% probability (63/144); that is, of the one hundred forty-four reported miRNAs targeting host mRNAs, only sixty-three have been found to repress viral replication. Therefore, it is necessary to consider the target type during antiviral exploration of miRNA.

### miRNAs targeting the 3’ UTR or ORF of viral mRNAs exhibit a higher antiviral ratio than miRNAs targeting the 5’ UTR of viral mRNAs

Apart from the target type, the target site of miRNA in a given viral mRNA may also notably influence the regulatory effect. As shown in Table 1, the miRNAs targeting the ORF of a viral mRNA showed the highest probability (96.43%) of inhibiting viral infection, followed by miRNAs targeting the 3’ UTR of a viral mRNA, which exhibited an 80.95% probability of inhibiting viral infection. Unfortunately, the antiviral ratio of miRNAs targeting the 5’ UTR of viral mRNAs is only 42.86%. Thus, it is inadvisable to select miRNAs targeting the 5’ UTR of a viral mRNA in the initial stage of antiviral agent exploration.

Overall, miRNAs targeting the 3’ UTR or ORF of viral mRNA are ideal candidates for the exploration of antiviral agents, and it is advisable to select these miRNAs as study materials at the beginning of an investigation into miRNAs as antiviral agents against viruses. However, the studies presented in Supplementary Table S1 are based mainly on the complementary base pairings between miRNAs and target mRNAs, and insufficient consideration has been directed to the biology of the viruses. For the viruses discussed in this paper, i.e., HCoVs, some special issues corresponding to their molecular biology need to be considered, which may make the exploration into anti-HCoVs miRNAs unique.

## Specific interaction modes of miRNAs-HCoVs corresponding to HCoV molecular biology

### The HCoV genome may be directly targeted by miRNAs upon viral entry into host cells

**Figure 1:**
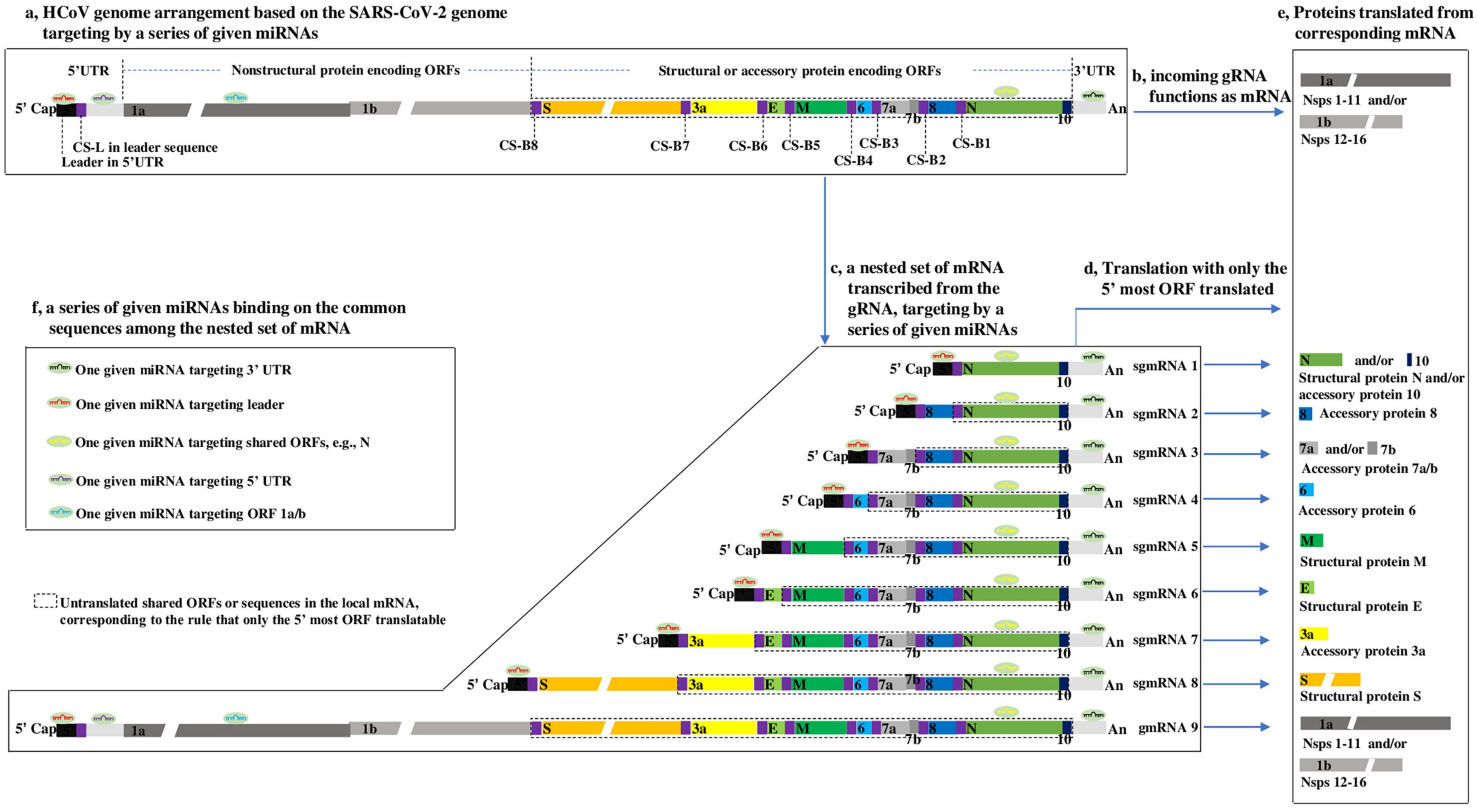
Outline showing HCoV genome arrangement, nested set of mRNA (Thiel et al., 2003; Zuniga et al., 2004), translation strategy (Sola et al., 2015), and proposed interaction modes of miRNAs-HCoV mRNAs, based on the SARS-CoV-2 genome (Wu et al., 2020). (a) HCoV genome arrangement. The SARS-CoV-2 genome consists mainly of a 5′ cap, a leader, the 5’ UTR, 12 ORFs, 9 CSs within TRS-L or TRS-B, 3’ UTR, and poly (a), arranged according to the aforementioned order. ORFs 7b and 1b are highlighted to indicate that they overlap with ORF 7a and ORF 1a, respectively. The gaps in the ORF 1a, ORF 1b and S indicate that their lengths are presented on different scales due to space limitations. To clearly illuminate the transcription of genome described in Supplementary Figure S3, the lengths of CS-Bs and CS-L are highlighted but do not correspond with their actual lengths, which are proposed to be ~6 nucleotides long. (b) Genomic RNA (gRNA) functions as mRNA. Upon HCoVs entering host cells, the first products synthesized are the nsps from ORF 1a/b in the viral gRNA (Lai and Cavanagh, 1997) when the gRNA can be directly targeted by miRNAs. (c–e) The nested set of HCoV mRNA and their Translation strategy. In most cases, only the 5′ most ORF is translated into a protein. The untranslated sequences or ORFs are included in the dotted line. The boxes within the same color refer to the same or complementary sequences or their translated proteins. (f) The proposed interaction modes of miRNAs-HCoV mRNAs. Because of the common sequences shared by the nested set of mRNAs, for a given HCoV, one given microRNA has the potential to target one, many or all viral mRNAs.

Common to all CoVs, the genome is arranged in the order 5′ cap-leader sequence-5’ UTR-ORF 1a-ORF 1b-S-E-M-N-3’ UTR-poly(A) (Plant and Dinman, 2008; Hartenian et al., 2020). The leader is a segment of the 5’ UTR, given its importance, which is separately listed here. ORFs 1a and 1b are two overlapping genes encoding nonstructural proteins (nsps) and encompass nearly two-thirds of the genome. S, E, M, and N are four basic ORFs encoding corresponding structural proteins and constitute one-third of the 3′ genome. In addition, there is a special sequence, called the translation-regulating sequence (TRS), at the 3′ end of the leader and preceding each ORF, termed TRS-L and TRS-B, respectively. TRS-L and TRS-B include a highly conserved core sequence (CS) with high homology, termed CS-L and CS-B, respectively. Furthermore, a variable number of ORFs encoding virus-specific accessory proteins are interspersed among the four basic ORFs (Figure 1a).

The CoV genome is a positive-sense, single-stranded RNA (Baltimore, 1971), and the size ranges from 27 ~ 30 kb in length, making it one of the largest known viral genomes (Hartenian et al., 2020). The nature of the CoV genome, approximately 30 kb, is not remarkable, but its type, consisting of positive-sense, single-stranded RNA, is surely considered from the perspective of miRNA regulation. This type of genomic RNA (gRNA) is not only infectious but can also function as mRNA-translating nsps 1–16, i.e., protease-related proteins (nsps 1–11) and polymerase-related proteins (nsps 12–16; Lai and Cavanagh, 1997; Figure 1b), which means that the gRNA can be directly targeted by miRNAs when it functions as mRNA translating proteins (Figures 1a,f). Upon CoVs entering host cells, the first products synthesized are the nsps from ORF 1a/b in the viral gRNA (Lai and Cavanagh, 1997; Figure 1b); only then can the virus proceed to the next life cycle. Hence, ORF 1a/b mRNA in CoVs is identical to the genome in sequence and size. Theoretically, the HCoV genome may be targeted and inhibited by miRNAs when it functions as a mRNAs translating nsps. In-depth interactions between miRNAs and gRNA (ORF 1a/b) are discussed in the next section.

### A common 5′ leader and 3’ UTR in the nested set of viral mRNAs may facilitate the antiviral effect of miRNAs on HCoVs

The CoV transcriptome is a nested set of subgenomic mRNAs (sgmRNAs) generated from the gRNA (Lai and Cavanagh, 1997; Plant and Dinman, 2008; Artika et al., 2020; Figure 1c). During the transcription of the nested set of mRNAs from gRNA, the CS is a key element, and the most acceptable model of transcription of the nested set of mRNAs has been repeatedly described (Thiel et al., 2003; Zuniga et al., 2004). To facilitate reading of the paper, the model is depicted in Supplementary Figure S3. From the nested set of mRNAs (Figure 1c), it can be inferred that:

(1) The CoV transcriptome consists of a nested set of sgmRNAs with a sequence identical to that of the gRNA. The longest mRNA is gRNA (refer to gmRNA 9 for SARS-CoV-2), transcribed throughout from the 3′ end to the 5′ end of gRNA, and the smallest mRNA is sgmRNA 1, transcribed from the 3′ end to CS-B1 (Supplementary Figure S3).

(2) All CoV mRNAs share a common 5′ leader and 3’ UTR (Lai and Cavanagh, 1997). Significantly, the common 5′ leader and 3’ UTR sequences in the nested set of mRNAs may be quite significant in that the commonalities suggest that for a given HCoV, a miRNA with the ability to bind to the 5′ leader or 3’ UTR may be able to target each mRNA, dramatically increasing the antiviral effect on the HCoV (Figures 1c,f). This is an advantage of exploring miRNAs as antiviral agents against HCoVs.

(3) The longer mRNA includes all the sequences in the shorter mRNA; therefore, the ORFs in the smallest mRNA (e.g., N) are present in all other longer mRNAs; alternatively, the longest mRNA (i.e., gmRNA 9) shares all the ORFs in the remaining shorter sgmRNAs 1–8. The regulatory roles of miRNAs by targeting a shared ORF (e.g., N) may be complicated due to the translation pattern of CoV mRNA (described in the next section). In addition, ORF 1a/b and 5’ UTR (except 5′ leader) are present only in gmRNA 9, which also makes sense for the regulation of miRNAs as follows.

### ORF 1a/b is proposed to hold greater certainty as a miRNA target than the shared ORFs in the nested set of HCoV mRNAs

Generally, animal mRNA always only has an ORF, with a start codon and a stop codon, namely, monocistronic. In contrast, CoV mRNA is structurally polycistronic, including one more ORF (Figure 1c); however, CoV mRNA is always functionally monocistronic, with only the first ORF relative to the 5′ end expressed (Figures 1d,e). This translation pattern is a result of CoV proteins translating in a canonical 5′-cap-dependent manner, in which the nucleotide chain (mRNA) has to enter ribosomes from the 5′-cap to initiate translation at the start codon and stop at the stop codon in the first ORF near the 5′ end (Plant and Dinman, 2008). There are a few cases in which some ORFs are translated in a variety of 5′-cap-dependent or-independent mechanisms (Kozak, 1989; Liu and Inglis, 1992; Fischer et al., 1997; Senanayake and Brian, 1997; Baranov et al., 2005), but they cannot change the overall knowledge about the translation pattern of CoV mRNAs. On the basis of the CoV mRNA translation pattern, the following can be inferred:

(1) An ORF in a shorter mRNA is shared by other longer mRNAs but only translated in the shorter mRNA, where it lies most near the 5′ end. In other words, although a longer mRNA shares the ORFs of shorter mRNAs, the shared ORFs are usually untranslated in longer mRNAs. For example, N is shared in all mRNAs, while it is only translated in sgmRNA 1 (called the “translated part” here) and untranslated in sgmRNAs 2–8 and gmRNA 9 (called the “untranslated part”), as indicated in Figures 1d,e.

From the characteristics described above, it is suggested that for a miRNA with a target site in a certain shared ORF, the untranslated part may reduce the repression effect of miRNA on the translated part by competitively binding to the miRNA, reducing the level of miRNA binding to the translated part. Taking a miRNA targeting the N as an example, the miRNA is thought to repress the translation of N in sgmRNA 1, where the N is translatable; however, this repression may be relieved by the untranslated N in sgmRNA 2–8 and gmRNA 9, because the untranslated N in these mRNAs might bind to the miRNA, competitively reducing the level of miRNA binding to the translated N in sgmRNA 1. In addition, no evidence has reported that base pairing between the untranslated N and miRNA has a repressive effect on the translation of sgmRNAs 2–8 and gmRNA 9, where the N is untranslatable. Overall, in the nested set of CoV mRNAs, whether the base pairing between untranslated ORFs and miRNAs plays a repressive effect on local mRNAs (where the untranslated ORF is located) is unknown; however, base pairing may decrease the repressive effect of miRNAs on the corresponding translated part. Thus, it is unadvisable to select miRNAs targeting the shared ORFs in the nested set of HCoV mRNAs as study materials at the beginning of an investigation into miRNAs as antiviral agents against HCoVs. It is important to explore whether the base pairing between untranslated ORFs and miRNAs has a regulatory effect on local mRNAs.

(2) ORF 1a/b is present only in the longest CoV mRNA; therefore, ORF 1a/b may be an appropriate target for miRNAs, and it can be efficiently targeted by a given miRNA without the influence of the foregoing base pairing of untranslated ORFs and miRNA. More importantly, as described above, upon entering cells, the first molecule synthesized by HCoVs is the replicase-related proteins encoded by ORF 1a/b in the incoming gRNA (Figure 1b). Thus, the repression of ORF 1a/b translation by a given miRNA may be an effective and important mechanism for interrupting the HCoV life cycle. In contrast, repressing the translation of other ORFs, such as S, E, M, and N, may be less effective because the effect may be partially counteracted by the shared untranslated ORF in the nested set of mRNAs. Thus, ORF 1a/b may hold greater promise as a miRNA target than the shared ORFs in the nested set of HCoV mRNAs.

To clearly pinpoint the advantages and possible pitfalls of exploring a given miRNA as an antiviral agent against HCoVs, a series of given miRNAs classified by the location of their target site (s) in the HCoV genome, along with their characteristics of antiviral effects, are summarized in Table 2, based on the foregoing general and specific interaction modes of miRNA-HCoV. The miRNAs targeting the 3’ UTR or ORF 1a/b in the HCoV genome are listed as the first two best candidates for the exploration of antiviral agents (Table 2). Table 2 will provide intuitive guidance for research into the exploration of miRNAs against HCoVs.

**Table 2:** The characteristics of antiviral effects mediated by a series of miRNAs targeting different regions of the HCoV genome, reported on the basis of the SARS-CoV-2 genome.

Location of target site of a given miRNA*	mRNA targeted^†^	Speculated strength of antivirus effect^‡^	Pitfalls	Possibility of antivirus (%)^§^	Priority level^‖^
Leader (including CS-L)	sgmRNAs 1–8, gmRNA 9	⭐⭐⭐⭐⭐⭐⭐⭐⭐	The possibility of inhibiting infection is low	42.86	11
5’ UTR (not including leader)	gmRNA 9	⭐	The possibility of inhibiting infection is low	42.86	12
ORF 1a/b	gmRNA 9	⭐	Not recognized	96.43	2
S	sgmRNA 8, gmRNA 9	⭐⭐	(1) untranslated S in gmRNA 9 may bind to the given miRNA, decreasing repression effect of the miRNA on translated S in sgmRNA 8; (2) the regulatory role of the untranslated binding on the local mRNA is unknown.	Unknown	10
ORF 3a	sgmRNAs 7–8, gmRNA 9	⭐⭐⭐	(1) Untranslated 3a in sgmRNAs 8 and gmRNA9 may bind to the given miRNAs, decreasing repression effect of the miRNA on translated 3a in sgmRNA 7; (2) The regulatory role of the untranslated binding on the local mRNA is unknown	Unknown	9
E	sgmRNAs 6–8, gmRNA 9	⭐⭐⭐⭐	(1) Untranslated E in sgmRNAs 7–8 and gmRNA 9 may bind to the given miRNA, decreasing repression effect of the miRNA on translated E in sgmRNA 6; (2) The regulatory role of the untranslated binding on the local mRNA is unknown	Unknown	8
M	sgmRNAs 5–8, gmRNA 9	⭐⭐⭐⭐⭐	(1) Untranslated M in sgmRNAs 6–8 and gmRNA 9 may bind to the given miRNA, decreasing repression effect of the miRNA on translated M in sgmRNA 5; (2) The regulatory role of the untranslated binding on the local mRNA is unknown.	Unknown	7
ORF 6	sgmRNAs 4–8, gmRNA 9	⭐⭐⭐⭐⭐⭐	(1) Untranslated 6 in sgmRNAs 5–8 and smRNA 9 may bind to the given miRNA, decreasing repression effect of the miRNA on translated one in sgmRNA 4; (2) The regulatory role of the untranslated binding on the local mRNA is unknown	Unknown	6
ORF 7a/b	sgmRNAs 3–8, gmRNA 9	⭐⭐⭐⭐⭐⭐⭐	(1) Untranslated 7a/b in sgmRNAs 4–8 and gmRNA 9 may bind to the given miRNAs, decreasing repression effect of the miRNA on translated one in sgmRNA 3; (2) The regulatory role of the untranslated binding on the local mRNA is unknown	Unknown	5
ORF 8	sgmRNAs 2–8, gmRNA 9	⭐⭐⭐⭐⭐⭐⭐⭐	(1) Untranslated ORFs 8 in sgmRNAs 3–8 and gmRNA 9 may bind to the given miRNAs, decreasing repression effect of the miRNA on translated one in sgmRNA 2, and the regulatory role of the untranslated binding on the local mRNA is unknown	Unknown	4
N or ORF 10	sgmRNAs 1–8, gmRNA 9	⭐⭐⭐⭐⭐⭐⭐⭐⭐	(1) Untranslated N/10 in sgmRNAs 2–8 and gmRNA 9 may bind to the given miRNAs, decreasing repression effect of the miRNA on translated one in sgmRNA 1; (2) The regulatory role of the untranslated binding on the local mRNA is unknown	Unknown	3
3’ UTR	sgmRNAs 1–8, gmRNA 9	⭐⭐⭐⭐⭐⭐⭐⭐⭐	Not recognized	80.95	1

SARS-CoV-2 has 9 mRNAs in all, namely, sgmRNAs 1–8 and gmRNA 9. *The location of the target site corresponds to a region from the 5′ to 3′ end of the SARS-CoV-2 genome. ^†^The HCoV mRNAs targeted by the same miRNA, which share the same target sequence. ^‡^The speculated strength of antivirus effect according to the number of target mRNAs, based on the premise that the given miRNA represses the translation of the targeted mRNA. ^§^The possibility of the antiviral effect of a given miRNA is determined from the statistical analysis summarized in Table 1. ^‖^Refers to the priority level of the given miRNA candidate as the object of further research; this rank is determined by the number of mRNAs targeted, predicted strength of the antiviral effect, potential pitfalls in identifying the target, and possibility of antiviral effect. For a given miRNA, the smaller the number is, the higher the priority level is.

### The subcellular compartmentalization of HCoV mRNAs during the viral life cycle is accessible to miRNAs

**Figure 2:**
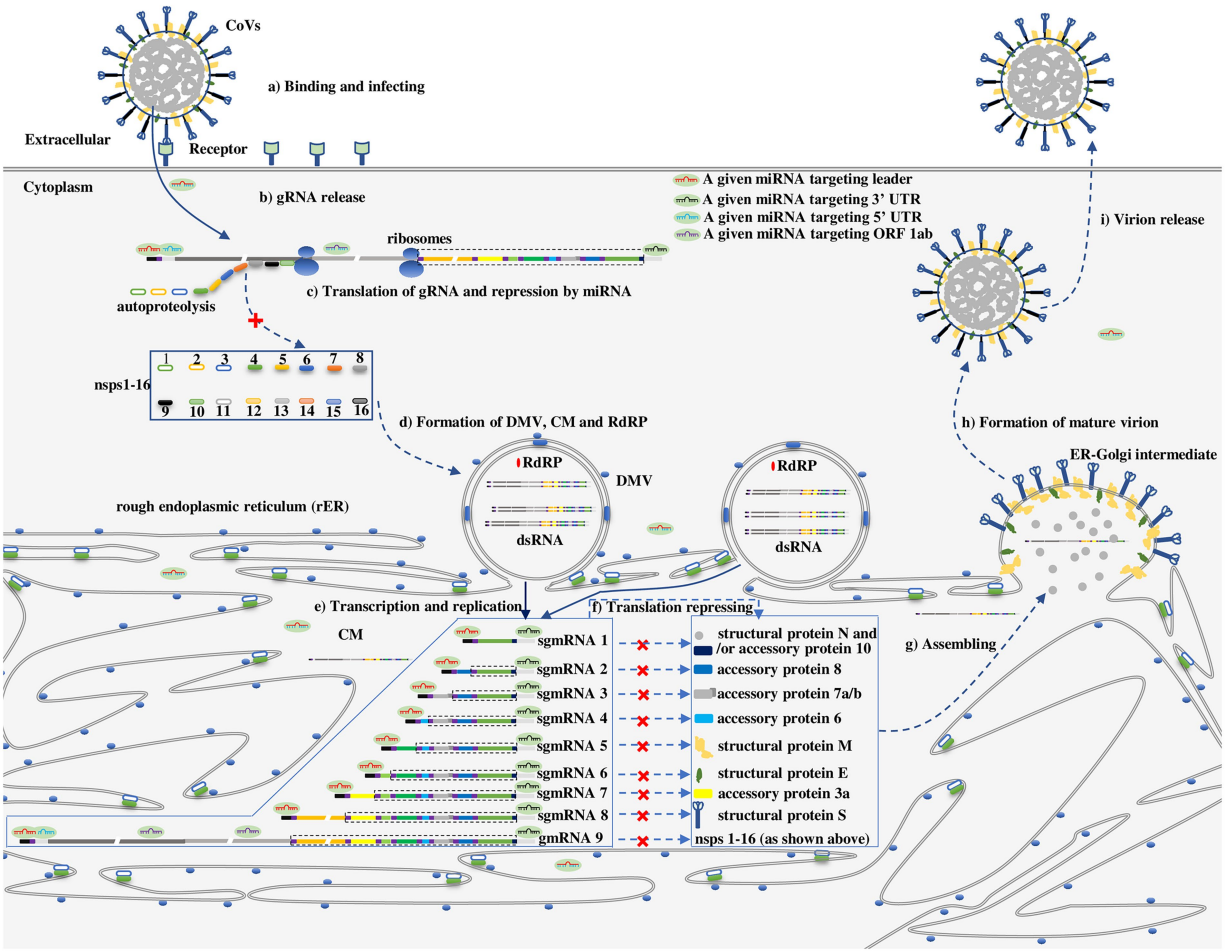
The proposed repressive effect of a series of given miRNAs on HCoVs in different subcellular compartments along with the viral life cycle (Hartenian et al., 2020). (a) Binding and infecting: CoVs bind to a cell receptor and invade cells *via* either cytomembrane fusion or endocytosis. (b) gRNA release: gRNA is released and uncoated, enabling its recognition by ribosomes. (c) Translation of gRNA and repression by miRNAs in unremodeled cytosol: Upon release of gRNA, nsps1-16 are translated from ORF 1a/b in gRNA, however, the process can be repressed by a miRNA targeting a leader, ORF 1a/b, 3’ UTR. (d) Formation of double-membrane vesicles (DMVs), convoluted membranes (CMs) and RdRPs: RdRPs, DMVs and CMs are assembled or established by nsps1-16. (e) Transcription and replication: Replication and transcription of gRNA in DMVs to generate a nested set of subgenomic mRNAs (sgmRNAs) or gmRNAs. (f) Translation of the nested set of HCoVs mRNA and repression by miRNAs on CMs or in CMs-protected cytosol: The nested set of mRNAs is translated into corresponding proteins on CMs or in CMs-protected cytosol. However, the translation of mRNA can be repressed by a miRNA targeting a leader, ORF 1a/b, or 3’ UTR. (g) Structural proteins and gRNA assembly in the ER-Golgi intermediate leading to (h) formation of mature virions (i) that are released from a cell. As in Figure 1c, the untranslated sequences or ORFs in the sgmRNA are indicated by dotted lines.

The general life cycle of HCoVs is depicted in Figure 2 (Hartenian et al., 2020). For HCoVs, the first molecule synthesized upon entering cells (Figure 2a) is a replicase protein (nsps1-16) encoded by ORF 1a/b in the incoming gRNA (Figures 2b,c), which is essential for continuation to the next CoV life cycle stage, e.g., rearrangement of intracellular membranes (Figure 2d) and the expression of structural proteins S, E, M and N (Figures 2e,f; Hartenian et al., 2020). It is a consensus that the proteins essential for internal cellular activities are translated in the cytosol by free ribosomes, whereas proteins exported from the cell with functions outside the cell are mainly translated on the rough endoplasmic reticulum (rER) by membrane-bound ribosomes (Johnson et al., 2001). On the basis of this premise, the replicase proteins of HCoVs critical for the internal cellular synthesis of gRNA and structural proteins have been proposed to be translated in the cytosol (Figure 2c), while the synthesis of structural proteins that are exported out of cells as viral particles has been proposed to take place on the cellular membrane (Figure 2f).

However, the subcellular compartmentalization where HCoV mRNAs are translated is slightly more complicated because viruses rearrange intracellular membranes (Stapleford and Miller, 2010; Figure 2d). Similar to many other positive-sense RNA viruses, HCoVs typically manipulate intracellular membranes to create a membrane-protected microenvironment for viral genome transcription and translation that does not trigger host immune system sensing, and individual viruses show specific membrane selectivity among the membranes derived from multiple organelles, including the rER, Golgi apparatus, endosomes, lysosomes, peroxisomes, chloroplasts, mitochondria, and vacuoles, as proven by tomographic electron microscopy (Stapleford and Miller, 2010). Facilitated by nsp 3, nsp 4, and nsp 6, partial products translated from ORF 1a/b during initial protease-related protein synthesis after entering cells, HCoVs preferentially hijack and reform the rER membrane to generate double membrane vesicles (DMVs), convoluted membranes (CMs), and ER-Golgi intermediate compartments (Figure 2d) that are involved in the transcription of the viral genome, translation of viral mRNA and assembly of virion particles, respectively (Figures 2e–g; Hartenian et al., 2020; Wang et al., 2022). DMVs and CMs are interconnected to each other and contiguous with the rER. It has been proposed that the translation of HCoV mRNAs takes place mainly in newly formed CMs (Wang et al., 2022). Thus, HCoV mRNA is translating in one of three possible subcellular compartments: (1) in unremodeled cytosol, ORF 1a/b is translated into a replicase protein in the early stage of infection; (2) in the membrane-protected cytosol, ORF 1a/b is translated into a replicase protein after the establishment of CMs; (3) on CMs, ORFs encoding structural proteins (e.g., S, E, M, N) are translated after establishment of CMs (Figure 2f). The three subcellular compartments where HCoV mRNAs are thought to be translated have been theoretically identified (Klein et al., 2020; Wang et al., 2022).

Whether the three compartments where HCoV mRNA functions are accessible to miRNAs is a key factor in determining the effect of miRNAs on mRNAs. Functional miRNAs in the miRISC diffuse throughout the cytosol; therefore, the miRNA readily regulates the translation of ORF 1a/b in the free cytosol in the early stage of infection (Figure 2c). In addition, miRNAs have been detected in multiple subcellular compartments, including the rER (O’Brien et al., 2018). These observations suggest that miRNAs can be transported through or accumulate on CMs, playing a regulatory role in the translation of CoV mRNA in CMs (Figure 2f). Combined with the CoV molecular biology (Hidalgo et al., 2021) and interaction modes of miRNAs and viruses (Kim et al., 2009; Zhang et al., 2012; Gebert and MacRae, 2019; Zhou et al., 2020), the repressive effect of miRNA on the CoV life cycle is proposed in Figure 2.

## Discussion

In this review, the action modes of miRNAs and viral biology are conjointly considered for the first time. Many important findings on the general and specific interaction modes of miRNAs-HCoVs corresponding to their molecular biology are found and discussed. Combining all the findings, we show that miRNAs exhibit two meaningful advantages, making them worthy of exploration as potential antiviral agents against HCoVs. First, miRNAs targeting the 3’ UTR, 5′ leader, or ORF 1a/b have the potential to inhibit HCoV translation in the early and middle stages of infection (Figures 2c,f). Second, for a given HCoV, one certain miRNA may target multiple, even all viral mRNAs, by binding to the 3’ UTR, 5′ leader sequence or some other shared sequences, signifying tremendous antiviral effect (Figure 2f).

In addition, the review finds an academic gap in the regulatory role of miRNAs on targets. For a given HCoV, the nested set of mRNAs shares many common ORFs (Figure 1c), part of which are untranslatable, with only the 5’most ORFs translated. However, no evidence has reported that base pairing between the untranslated ORFs and miRNA plays a regulatory effect on the local mRNAs where the untranslated ORFs are located (Figure 1c). Importantly, the unknown effects of base pairing between the shared untranslated ORFs and miRNAs on the local mRNAs, as explained herein, may complicate the exploration of miRNAs as potential antiviral agents against HCoVs. To simplify the complexity, a series of miRNAs classified by the location of their target sites in the HCoV genome, along with their characteristics of antiviral effects, are summarized in Table 2. Many miRNAs have been proposed to directly target different regions of the HCoV genome (Khan et al., 2020; Kim et al., 2020; Sardar et al., 2020; Sato et al., 2021), from which Table 2 can provide intuitive guidance for selecting miRNAs with higher CoV mRNA affinity and greater likelihood of antiviral effects as candidates for further exploration. With the aid of Table 2, researchers can design and synthesize specialized miRNAs targeting a preferred target site in the HCoV genome for further exploration of antiviral agents against HCoVs (Wakabayashi et al., 2019).

Unfortunately, the experimental evidence is too limited, and the specific interaction modes of miRNAs-HCoVs are theoretically proposed, largely based on the action mode of miRNA and HCoV molecular biology; hence, they need to be further validated. Apart from the foregoing biological characteristics, other factors need to be taken into consideration for a miRNA to achieve binding to potential targets, including the accessibility of miRNA target sites (Kim et al., 2006; Akhtar et al., 2019), AGO-specific miRNAs (Liu et al., 2004; Meister et al., 2004), the free energy involved in complementary base pairing, the conservativeness of target sites in different species, and so on. For example, senior structures of RNA are pervasive throughout viral genomes (e.g., 5′ leader, 3’ UTR) and have important effects on replication, protein synthesis, packaging, evasion of host immune factors, and the hijacking of host cell machinery (Boerneke et al., 2019). However, senior structures of RNA may influence the accessibility of a miRNA to its target, especially the target located in the senior structures, which means that theoretical interaction between a certain miRNA and target sequence may not happen. Therefore, how to identify miRNAs that achieve binding to their targets (refer to HCoV RNA here) is a key problem during the exploration of miRNAs as antiviral agents. Fortunately, recent technologies of molecular biology allow researchers to overcome the foregoing problem. The technology is termed “CLASH or HITS-CLI” (Helwak and Tollervey, 2014; Moore et al., 2015), by which the endogenous AGO-miRNA–mRNA complexes are first cross-linked/fixed using UV 254 nm, and then the AGO-bound RNAs, i.e., miRNA and targeted mRNA, are covalently ligated into hybrids, extracted with immunoprecipitation of AGO, and sequenced. The method can detect the miRNA interactome to survey miRNAs binding to their target sites, and it has been successfully used to identify many miRNAs binding to the RNA of 15 different viruses (Scheel et al., 2016). CLASH may help researchers identify miRNAs with different target sites in a given HCoV genome, e.g., the 5′ leader, ORF 1a/b, and 3’ UTR, with which researchers can conduct the following experiments to verify the specific interaction modes of miRNAs-HCoVs.

Subsequently, the safety of miRNA-based products becomes a major concern when an effective miRNA agent against a certain HCoV is successfully explored. The inhibition efficiency of a miRNA partially depends on the molar ratio between the miRNA and its target genes. Generally, the number of copies of a cellular endogenous miRNA is impossible to match with the exponential growth of RNA viruses and their genome. Therefore, it is necessary to modify the level of the miRNA *in vivo* by using exogenous miRNAs as antiviral agents, which may change host gene expression, leading to concerns about adverse effects. To date, miRNA-based drugs have been reported to be safe in clinical trials (Sanghani et al., 2022); nevertheless, safety concerns are inevitable because of the special complementary approach between miRNAs and their targets. To minimize the potential side effects of miRNAs as much as possible, the paper focuses on the miRNA targeting the HCoV gene, not the host gene, in the imagine that the former miRNA may have less possibility to induce side effects. For a miRNA set to inhibit virus infection by directly targeting viral genes, the lower the number of targeted host genes, the safer the miRNA. Detecting the miRNA interactome *via* CLASH in the context of virus infection may help researchers to identify such a miRNA with fewer targeted host genes. There may be many other obstacles and concerns for the exploration of miRNAs as antiviral agents; however, the exploration of miRNAs as antiviral agents against HCoVs may be feasible according to the findings in the review and will be worthy in the context that severe respiratory diseases caused by HCoVs have periodically emerged in the last two decades.

In conclusion, from the perspective of molecular biology, the intrinsic nested set of HCoV mRNAs may make miRNAs promising antiviral agents against HCoVs because these kinds of mRNAs share many common sequences and may be targeted by the same miRNA. However, the findings need to be experimentally validated.

## Author contributions

TX: conceptualization, formal analysis, and writing-original draft. TX, YJ, QW, L-xL, WZ, and XZ: data curation. BZ and XL: writing-review and editing. All authors contributed to the article and approved the submitted version.

## Funding

This work was supported by Jiangxi Provincial Department of Education (no. GJJ211212), National Natural Science Foundation of China grant (no. 82141214), Jiangxi Provincial Administration of Traditional Chinese Medicine (no. 2020A0312) and Jiangxi University of Chinese Medicine Science and Technology Innovation Team Development Program (CXTD22011) to TX.

## Conflict of interest

The authors declare that the research was conducted in the absence of any commercial or financial relationships that could be construed as a potential conflict of interest.

## Publisher’s note

All claims expressed in this article are solely those of the authors and do not necessarily represent those of their affiliated organizations, or those of the publisher, the editors and the reviewers. Any product that may be evaluated in this article, or claim that may be made by its manufacturer, is not guaranteed or endorsed by the publisher.
