# Supplementary material for: One microRNA has the potential to target whole viral mRNAs in a given human coronavirus
Source: Front Microbiol. 2022 Nov 10;13:1035044. doi: 10.3389/fmicb.2022.1035044 (PMC9686371; doi:10.3389/fmicb.2022.1035044)
Supplement: Supplementary file 1 [file Presentation_1.PDF]

One microRNA would be enough to target whole viral mRNAs in a given human coronavirus

Graphical Abstract

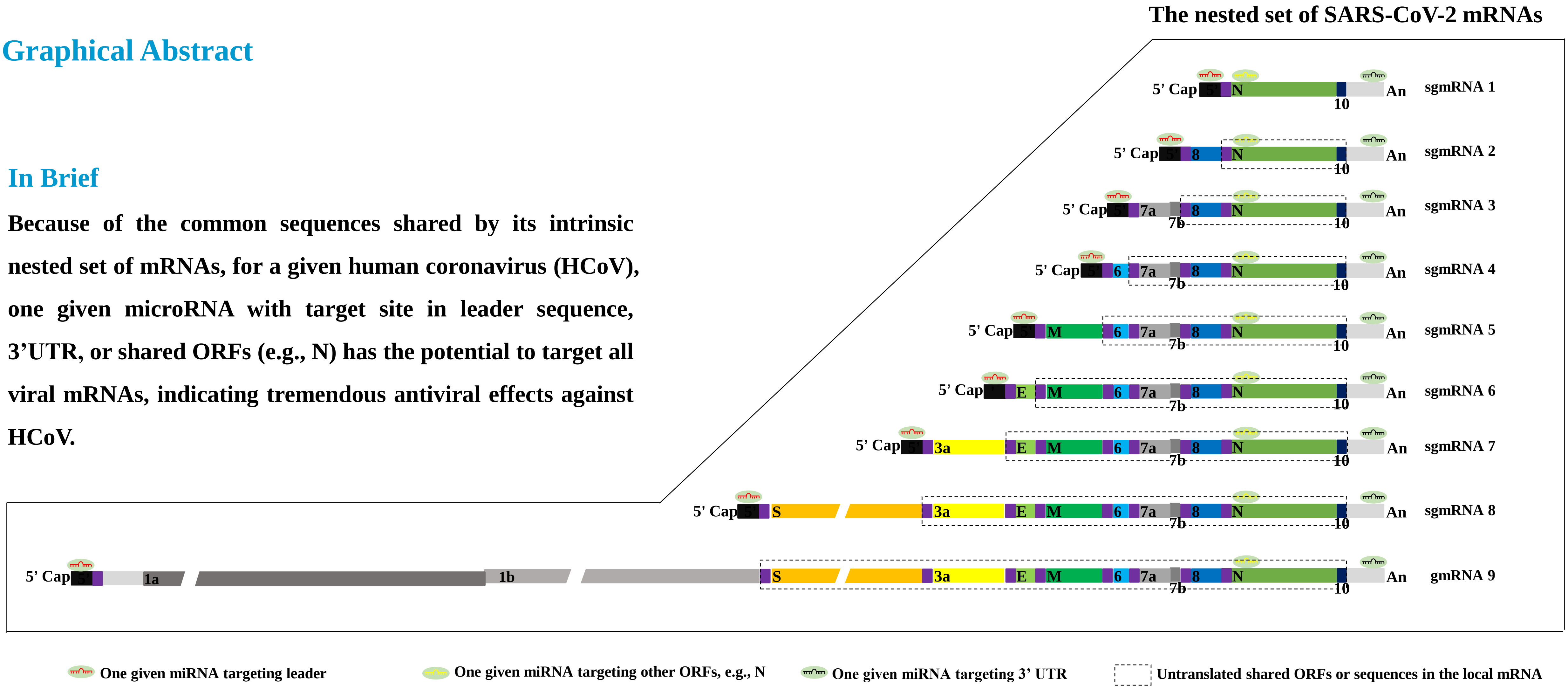

In Brief

Because of the common sequences shared by its intrinsic nested set of mRNAs, for a given human coronavirus (HCoV), one given microRNA with target site in leader sequence, 3'UTR, or shared ORFs (e.g., N) has the potential to target all viral mRNAs, indicating tremendous antiviral effects against HCoV.
